# Longitudinal Assessment of Strength, Functional Capacity, Oropharyngeal Function, and Quality of Life in Oculopharyngeal Muscular Dystrophy

**DOI:** 10.1212/WNL.0000000000012640

**Published:** 2021-10-12

**Authors:** Rosemarie H.M.J.M. Kroon, Johanna G. Kalf, Bert J.M. de Swart, Barbara M. van der Sluijs, Jeffrey C. Glennon, Vered Raz, Baziel G. van Engelen, Corinne G.C. Horlings

**Affiliations:** From the Departments of Rehabilitation (R.H.M.J.M.K., J.G.K., B.J.M.d.S.) and Neurology (B.G.v.E., C.G.C.H.), Donders Institute for Brain, Cognition and Behaviour, Radboud University Medical Center, Nijmegen; Department of Neurology (B.M.v.d.S.), Gelre Hospital Zutphen, the Netherlands; Conway Institute of Biomolecular and Biomedical Research (J.C.G.), School of Medicine, University College Dublin, Ireland; Department of Human Genetics (V.R.), Leiden University Medical Centre; and Department of Neurology (C.G.C.H., Maastricht University Medical Center, Maastricht, the Netherlands.

## Abstract

**Background and Objectives:**

Oculopharyngeal muscular dystrophy (OPMD) is a late-onset, progressive muscle disease. Disease progression is known to be slow, but details on the natural history remain unknown. We aimed to examine the natural history of OPMD in a large nationwide cohort to determine clinical outcome measures that capture disease progression and can be used in future clinical trials.

**Methods:**

Patients invited by their treating physicians or identified from the national neuromuscular database and invited family members were examined twice 20 months apart with fixed dynamometry; Medical Research Council (MRC) grading; maximum bite force and isometric tongue strength; Motor Function Measure (MFM); 10-step stair test; maximum swallowing, chewing, and speech tasks; and quality of life assessments.

**Results:**

Disease progression was captured by 8 of 18 measures over 20 months in 43 patients with genetically confirmed OPMD. The largest deterioration was seen in deltoid muscle strength (−27% [range −17% to −37%]), followed by the quadriceps (−14% [range −6 to −23%]), iliopsoas (−12.2%), tongue (−9.9%), and MRC sum score (−2.5%). The 10-step stair test (−12.5%), MFM part D1 (−7.1%), and maximum repetition rate of /pa/ (−5.3%) showed a significant decrease as well (all *p* < 0.05). The Physical Functioning domain of the Short Form-36 Health Survey significantly deteriorated (*p* = 0.044). No relationship was found between disease progression and genotype or disease duration (*p* > 0.05).

**Discussion:**

Despite the slow disease progression of OPMD, this study showed that several outcome measures detected progression within 20 months. Deltoid muscle strength, measured by fixed dynamometry, showed the greatest decline. These longitudinal data provide clinical outcome measures that can be used as biomarkers in future clinical trials.

Oculopharyngeal muscular dystrophy (OPMD) is an autosomal dominant, late-onset muscle disease. The estimated prevalence is ≈1:100,000, but several studies suggest that OPMD is underdiagnosed,^1-5^ mostly due to unfamiliarity among clinicians.^3^ The clinical characteristics of OPMD are ptosis due to weakness of the levator palpebrae muscle and dysphagia due to weakness of the pharyngeal muscles,^6^ both starting early in the disease course.^7^ Weakness of shoulder girdle and limb girdle muscles, even as an early sign,^8-10^ completes the clinical picture, but these symptoms are not always recognized as being part of OPMD, especially in the elderly.

No pharmacologic treatment is available for OPMD, but with recent developments in gene therapy, therapeutic trials in humans are forthcoming.^11-14^ Therefore, detailed information on the natural history and sensitive outcome measures to detect disease progression or therapy effect are urgently needed.^15^ Detailed analysis of the natural history will help us understand the variability in disease severity within families, which can possibly support therapy development. One small study including 8 patients with OPMD investigated disease progression over a period varying from 8 to 16 months with MRI of the muscles and the Motor Function Measure (MFM) and showed disease progression on the MRI but no disease progression on the MFM.^16^ No other longitudinal clinical studies of OPMD are available.

This study aims to examine in detail the natural history in a large nationwide cohort of patients with OPMD to determine clinical outcome measures that capture disease progression and can be used in future clinical trials.

## Methods

### Patients

Patients were invited by their treating physicians or identified from the national neuromuscular database (Computer Registry of All Myopathies and Polyneuropathies [CRAMP])^17^ in the Netherlands. Approximately 80 patients are known to be diagnosed with OPMD by a neurologist (source: CRAMP database). In addition, family members of the patients were asked to participate through an information letter. Thus, possible asymptomatic carriers and patients with subtle signs of OPMD could be included.

At baseline and at follow-up, all patients were interviewed to identify complaints related to oropharyngeal tasks (swallowing, chewing, and speaking) or to functioning of the limbs.

### Standard Protocol Approvals, Registrations, and Patient Consents

The study was approved by the local medical ethics committee (study NL54606.091.15), and all patients gave signed written informed consent.

### Clinical Examination

All patients were clinically examined at baseline and after ±20 months by the same investigator (R.H.M.J.M.K.). The measurements are explained below.

### Measurements of Muscle Strength

- Fixed dynamometry (Newtons) was performed with the strength transducer KAP-S 2 kN (Angewandte System Technik GmbH, Dresden, Germany) to measure the maximal isometric contraction of the shoulder abduction (deltoid muscle), hip flexion (iliopsoas muscle), and knee extension (quadriceps muscle). The best performance (maximum force) of the 3 measurements was used for analysis.
- Manual muscle testing was performed with the Medical Research Council (MRC) scale ranging from 0 (no muscle contraction) to 5 (maximal muscle strength)^18^ for neck extension and flexion, elbow extension and flexion, knee extension and flexion, hip abduction, hip flexion, foot dorsal flexion and foot plantar flexion, handgrip, wrist extension and flexion, and shoulder abduction bilaterally (score 0–130).
- Maximum bite force was measured with the Bite Force Gauge (Vrije Universiteit, Amsterdam, the Netherlands). The best performance of 3 trials was used for analysis.
- Maximum isometric tongue strength was assessed with the Iowa Oral Performance Instrument (model 2.3, IOPI Medical LLC, Woodinville, WA). Patients were instructed to push the bulb with the anterior part of the tongue against the roof of the mouth as hard as possible. The best performance of 3 trials was used for analysis.^19^

### Measurements of Functional Capacity

- The MFM consists of 3 parts: D1, concerning stance and transfer tasks; D2, concerning tasks of axial and proximal muscles; and D3, concerning tasks using distal muscles. The outcome ranges from 0% to 100%. A score of 100% implies no functional motor deficits.^20^
- A timed stair-walking test (10-steps) was measured in seconds.^21^ Patients were instructed to take the stairs as they normally would. The stairs had 1 handrail. The patients decided to hold the handrail or not.
- The Test of Masticating and Swallowing Solids (TOMASS) was performed by eating a cracker as fast as possible, measured in seconds.^22^
- The maximum swallowing capacity was measured by the maximum swallowing speed and maximum swallowing volume*.* For the maximum swallowing speed, patients were instructed to drink 150 mL water as quickly as possible.^23,24^ For the maximum swallowing volume, patients were instructed to swallow a maximal amount (milliliters) of water in 1 swallow.^25^
- The maximum speech capacity was measured by the maximum phonation time and maximum repetition rate.^26,27^ Maximum phonation time measures how long a patient can produce an /a/ in seconds. Norm values are available for several languages, also in Dutch.^28-30^ Maximum repetition rate is the number of syllables per second during the 5 first seconds (syllables per second) of producing the monosyllabic sequences /pa/, /ta/, /ka/ and a trisyllabic sequence /pataka/. The best score of 3 trials was used in the analysis.
- Videofluoroscopy of swallowing was performed with the Digital Swallowing Workstation (model 7120, Swallowing Signals Lab, KayPENTAX, Lincoln Park, NJ) to quantify the swallowing efficiency and safety using the Normalized Residue Ratio Scale (NRRS) and the Penetration Aspiration Scale. A detailed description of these measurements is given by Kroon et al.^31^
- Videotapes were made of eye movements and ptosis for offline analysis by one of the authors, an experienced neurologist (C.G.C.H). Ptosis was scored on both sides on a scale from 0 to 3 (0 = no ptosis, 1 = mild ptosis [above half of the pupil], 2 = severe ptosis [below half of the pupil], 3 = status after operative ptosis correction). Eye movements were scored as ophthalmoplegia present or absent.

### Quality of Life Scales

For an assessment of patients' quality of life, we used the International Quality of Life (INQoL) questionnaire and the Short Form-36 (SF-36) Health Survey.^32,33^

### Statistical Analysis

IBM SPSS Statistics (version 25; Armonk, NY) was used to conduct all statistical analyses, and values of *p* < 0.05 were considered statistically significant. The interviews and structured questionnaires were analyzed by counting the number of patients who reported any (worsening of) subjective complaint in any of the domains. Individual scores on the INQoL and SF-36 health assessment were calculated according to their requirements, and mean scores were estimated per domain. The results of all clinical measures at baseline and follow-up were compared by the use of paired-sample *t* tests to calculate the mean differences with their 95% confidence intervals (CIs). Differences of measures that were not normally distributed at baseline were also tested with the Wilcoxon signed-rank test. The mean differences and CIs were converted into percentages of worsening. The correlation between patient characteristics (disease duration and GCN-repeat size) and disease progression on all tasks was analyzed by calculating the Spearman ρ correlation coefficients.

### Data Availability

The anonymized data that support the findings of this study are available from the corresponding author on reasonable request.

## Results

### Patients

**Figure 1:**
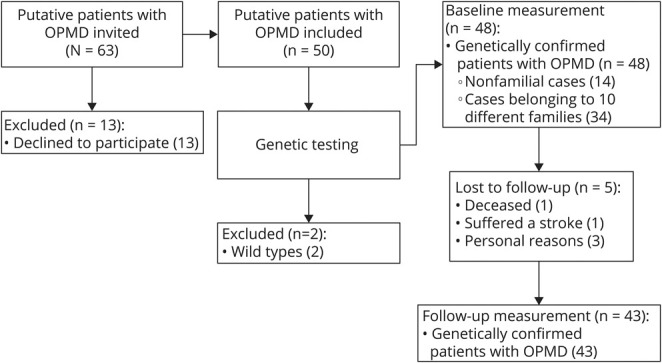
Flowchart of Participant Recruitment and Inclusion OPMD = oculopharyngeal muscular dystrophy.

Forty-four patients with OPMD were invited by their treating physicians or identified from the national neuromuscular database. Nineteen family members of these patients, who were therefore patients with putative OPMD, were asked to participate as well. Figure 1 shows the flowchart of participant recruitment and inclusion. After genetic testing, it appeared that 2 family members did not have a mutation in the *PABPN1* gene. Therefore, these 2 participants were excluded. The demographic and genetic details of the 43 patients with OPMD are summarized in Table 1. Four patients did not have any complaints at baseline; they are further referred to as asymptomatic carriers. The mean time between the 2 visits was 20 months (median 20 months, range 17–24 months).

**Table 1:**
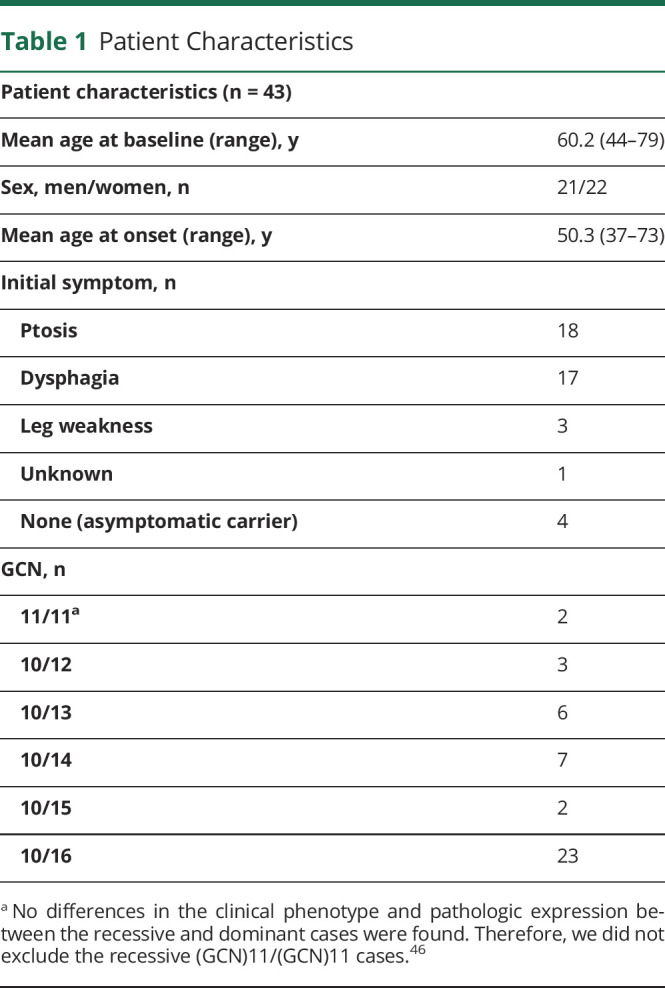
Patient Characteristics

At follow-up, 2 of 4 asymptomatic carriers reported subjective complaints and became symptomatic carriers; 1 individual reported “tired legs and difficulty climbing stairs,” and the other reported “a hoarse voice and difficulty with simultaneous tasks like eating and walking.”

### Clinical Measures

Nine of the 21 measures were not normally distributed, with 3 having a value of *p* < 0.05. However, when the Wilcoxon signed-rank test was used instead, the significance of the *p* values did not change. Table 2 shows the mean percentages of worsening on the 21 measures, of which 10 proved statically significant.

**Table 2:**
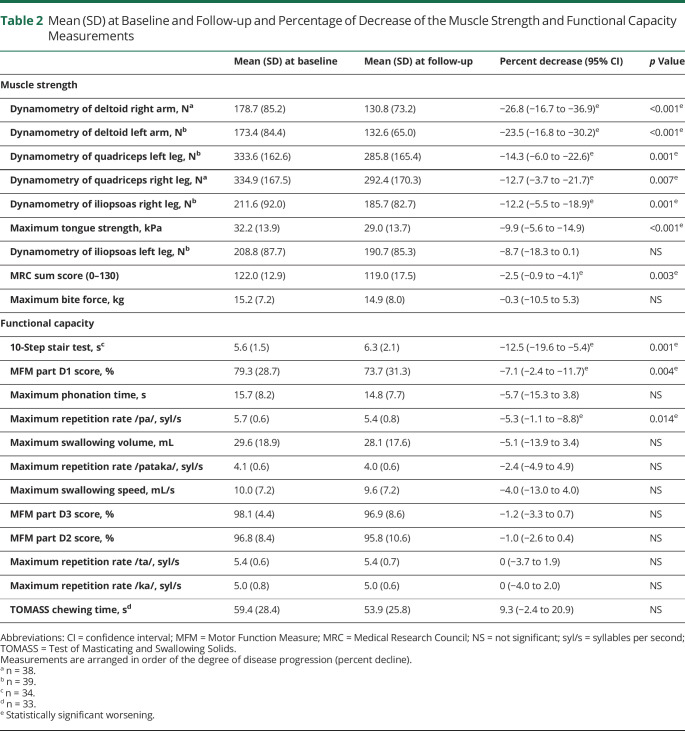
Mean (SD) at Baseline and Follow-up and Percentage of Decrease of the Muscle Strength and Functional Capacity Measurements

### Muscle Strength

#### Fixed Dynamometry

Fixed dynamometry of the deltoid muscle (left side), quadriceps (left side), and iliopsoas muscle (both sides) was performed in 39 patients. Thirty-eight patients underwent fixed dynamometry of the deltoid muscle and quadriceps (right side). Some patients were not able to perform the tasks due to injuries or technical errors.

**Figure 2:**
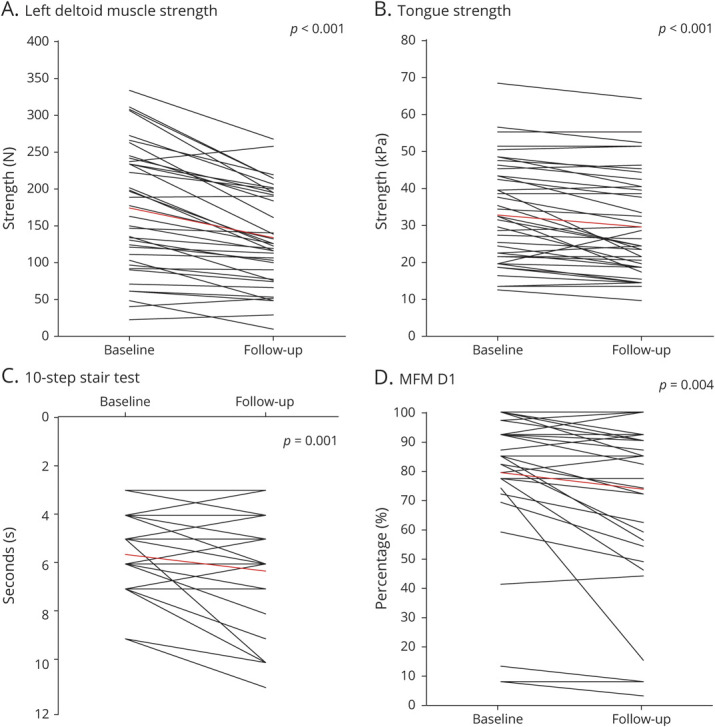
Measurements of Muscle Strength and Functional Capacity at Baseline and Follow-up (A) Muscle strength of the left deltoid muscle assessed with dynamometry, (B) tongue strength, (C) 10-step stair test, and (D) part 1 of Motor Function Measure (MFM). Each line represents a patient. Red line shows mean score.

All dynamometry measures showed a statistically significant decline over 20 months (Table 2, up to 27% decline), except for the iliopsoas muscle of the left leg (mean decline 8.7% [95% CI −18.3% to 0.1%]). The deltoid muscle showed the largest strength deterioration over time (Figure 2A, left arm mean decline 24% [95% CI −16.8% to −30.2%], right arm mean decline 27% [95% CI −16.7% to −36.9%]).

#### Maximum Isometric Tongue Strength

Maximum tongue strength showed a mean decline of 9.9% over 20 months (95% CI −5.6% to −14.9%, Table 2 and Figure 2B).

#### Manual Muscle Testing (MRC Scale)

The MRC sum score of all muscles showed a mean decline of 2.5% between baseline and follow-up (Table 2, 95% CI −0.9% to −4.1%). The MRC grading also showed a small but significant decrease of 0.1 to 0.2 points at follow-up for 3 individual muscle groups: hip adduction (both sides mean difference 0.19, *p* = 0.010), elbow flexion (right side mean difference 0.11, *p* = 0.024, left side mean difference 0.10, *p* = 0.044), and shoulder abduction (right side mean difference 0.14, *p* = 0.032, left side mean difference 0.14, *p* = 0.013).

### Functional Capacity

#### Stair-Walking Test

Thirty-four patients showed a mean decline of 12.5% (Figure 2C, [95% CI −19.6% to −5.4%]). Six patients at baseline and 9 patients at follow-up were not able to perform the stair-walking test due to severe muscle weakness in the legs.

#### Motor Function Measure

Part D1 of the MFM test, concerning stance tasks and transfers, showed a mean decline of 7.1% (95% CI −2.4% to −11.7%, Table 2 and Figure 2D). Parts D2 and D3 of the MFM did not show a significant change over 20 months (*p* > 0.05).

#### Swallowing, Chewing, and Speaking

Swallowing tasks (maximum swallowing speed and maximum swallowing volume) and chewing time did not worsen significantly during follow-up (Table 2). Five patients at baseline and 10 patients at follow-up were not able to perform the TOMASS chewing test. Of the speech capacity tests, only the maximum repetition rate of the syllable /pa/ was significantly slower at follow-up (mean decline 5.3% [95% CI −1.1% to −8.8%]).

#### Videofluoroscopy

Forty-two patients were able to perform the videofluoroscopy. The amount of abnormal pharyngeal residue of thin liquid (10 mL) in the valleculae increased significantly during follow-up (mean NRRS ratio 0.24 vs 0.13, *p* = 0.007). The NRRS ratios for the valleculae and pyriform sinus of the other consistencies did not show a significant difference between baseline and follow-up.

Patients showed no aspiration of thick liquids at baseline, while at follow-up, unsafe swallowing (Penetration Aspiration Scale score >3) was seen during swallowing 10 mL in 8% of the patients (*p* = 0.119) and 20 mL in 3% of the patients (*p* = 0.324). When patients swallowed 10 mL thin liquid, unsafe swallowing was less frequent at follow-up (baseline 21% of all patients, follow-up 10% of all patients, *p* = 0.033). No change in aspiration rate was seen for swallowing 20 mL of thin liquid and solid food.

#### Ptosis and Ophthalmoplegia

Ptosis analyses was done for 40 patients; measurements of 3 patients were missing because of poor video quality. At baseline, 31 patients had ptosis. Ptosis had worsened at follow-up for 3 patients: 1 patient went from no ptosis to mild ptosis, and 2 patients went from mild ptosis to severe ptosis. Seventeen patients underwent ptosis correction of the right or left eye at baseline; at follow-up, this increased to 19 patients.

Ophthalmoplegia analyses were performed in 37 patients; measurements of 6 patients were missing due to poor video quality. On the basis of the examination of 2 independent clinicians, subtle ophthalmoplegia was present in 14 of 37 patients at baseline and follow-up, especially in the upper vertical direction.

### Quality of Life Scales

The highest negative impact on quality of life was scored in the INQoL domains of muscle weakness, activities, body image, and fatigue (mean at follow-up 46.8, 37.1, 32.5, 29.6), but none of the domains of the INQoL showed a significant difference between baseline and follow-up. In the SF-36 Health Survey, the physical functioning domain showed a significant deterioration between baseline and follow-up (baseline mean [SD] 61.2 [30.9], follow-up 57.6 [31.4], *p* = 0.044).

### Asymptomatic Carriers

Each asymptomatic carrier scored somewhat lower on various muscle strength tasks (dynamometry of upper and lower extremities, maximum isometric tongue strength, and maximum bite force) and functional capacity tests (maximum swallowing volume and motor function measure).

### Natural History

The deltoid, quadriceps, and iliopsoas muscles showed the largest disease progression, followed by the tongue muscle (Table 2). Strength decline of these muscles was seen across all patients regardless of the disease severity, that is, in mildly and severely affected patients.

No relationship was found between patient characteristics (disease duration and repeat length [GCN]) and disease progression in muscle strength and functional capacity measurements (*p* > 0.05). Fast and slow rates of disease progression were found in asymptomatic and in severely affected patients.

## Discussion

Although disease progression in OPMD is known to be very slow,^7^ the main finding of this nationwide longitudinal cohort study is that deterioration can be detected by 8 different clinical measures, regardless of disease severity, during a period of only 20 months. In the absence of comparable studies, this implies that these measures (dynamometry of the deltoid, quadriceps, and iliopsoas muscles; maximum tongue strength; MRC sum score; 10-step stair test; part D1 of the MFM; and maximum speech repetition rate /pa/) could be used to detect disease progression within the time frame of a therapeutic trial.

With dysphagia being the main feature of OPMD, it is highly relevant that tongue strength is one of the features that significantly reduces over an average of 20 months with a mean of 10%. Indeed, the tongue muscle is the most affected oropharyngeal muscle in patients with OPMD as seen in muscle MRI measures.^34^ Moreover, tongue strength is easy to measure with commercially available handheld devices.^19^ However, the strength of the deltoid muscle showed an even greater mean decline over time (27%). This emphasizes that upper extremity involvement is an important and common feature of OPMD,^6^ and our results show that deltoid strength could be a valuable measure to detect disease progression or therapeutic effect in patients with OPMD.

Disease progression could also be measured by muscle strength measures of the lower extremities. Although some studies reported that the anterior thigh compartment is less affected on muscle MRI measures than the posterior part,^10^ in our dynamometry measurements, there was a significant decline in muscle strength of the quadriceps muscle over 20 months. This may suggest that the strength of the posterior compartment (i.e., hamstrings muscle) might be a sensitive measure of disease progression as well. In our study setup, however, dynamometry of the hamstrings was not possible, so this should be the subject of future studies.

The stair-walking test showed the largest decline of all functional capacity tests. In other natural history studies of slowly progressive neuromuscular disorders, for example, in facioscapulohumeral muscular dystrophy, the stairstep test did not show any clinical progression after 1 year.^35^ Furthermore, the MFM detected disease progression in our cohort, in contrast to an earlier study that showed no progression on the MFM in 8 patients with OPMD over a follow-up period of 8 to 16 months.^16^

Except for reduced tongue strength and maximum repetition rate of /pa/, no significant decrease was found in the functional capacity tests of chewing, swallowing, and speaking. Even maximum swallowing speed, which at baseline with a mean of 10 mL/s was already far below normal (25 mL/s),^31^ was only slightly and insignificantly reduced after 20 months. Apparently, it is not sensitive enough to detect a decline within 20 months, or swallowing is already so slow that further deterioration becomes less likely (bottom effect). During videofluoroscopy, only a few changes were seen. Unsafe swallowing occurred less frequently in those ingesting 10 mL thin liquid at follow-up, which may reflect compensatory actions preventing unsafe swallowing or a learned response between baseline and follow-up measurements. However, a small deterioration in unsafe swallowing was seen after ingestion of thick liquids. Thus, videofluoroscopy does appear to be a less appropriate technique to detect swallowing deterioration in OPMD over 20 months.

Typically, healthy people very slowly deteriorate on muscle-related tasks because of aging (normal age-related changes). Hence, in our previous work,^31^ we compared patients with OPMD with age-matched Dutch healthy controls on swallowing, chewing, and speaking tasks. Patients with OPMD scored significant lower on these tasks compared to age-matched healthy controls. In addition, for the stairstep test, healthy controls of approximately the same mean age (58.4 years) as our OPMD group showed much faster stairstep times compared to our OPMD group (mean per step 0.336 seconds vs 0.56 seconds).^36^ OPMD is suggested to be an accelerated aging disorder,^37,38^ so we expect that the disease progression on clinical outcome measures will be greater in patients with OPMD than in age-matched healthy controls. However, to the best of our knowledge, there are no longitudinal studies on the clinical outcome measures in healthy controls. The goal of this study was to detect changes on clinical outcome measures in patients with OPMD, which will be caused by disease progression and aging. A direct comparison to controls would explain this further but was not the scope of the current study.

At follow-up, 2 of the 4 asymptomatic carriers had become symptomatic. On an individual level, each patient scored lower on various measures at follow-up, and that measure corresponded to the subjective complaints of the patient (i.e., difficulty climbing stairs and tired legs). Larger cohorts of asymptomatic OPMD carriers may give more insights into the subtle signs of disease onset but are difficult to perform due to the rarity of the disease and the difficulty in finding asymptomatic carriers.

Future longitudinal studies with larger (international) cohorts can also observe the natural history of patients with OPMD per age group. In this study, analyses by age group were not possible due to the small number of cases per age. In addition, within-family analyses were not feasible in our study. Although 10 different families were identified within our study, 5 of them were very small (only 2 family members). Still, similarities between family members might overestimate the magnitude and precision of the estimated changes over time, suggesting that in future studies this should be taken into account as well. However, because OPMD is a rare disease, it is hard to perform large studies to allow such analyses.

According to the INQoL, the quality of life of our patients with OPMD was reduced by several factors, but mostly by muscle weakness and fatigue, which is in accordance with previous studies on the quality of life in patients with OPMD.^39,40^ Similar quality of life scores were also found in other populations with muscular dystrophies.^41^ Quality of life was not further reduced at follow-up except that patients' health status was slightly reduced according to the SF-36 Health Survey, but only in the physical functioning domain. This resonates with our main finding that captured disease progression on 8 of the strength and functional capacity measures. The overall quality of life scale does not seem to be useful in capturing progression within 20 months.

Some of the changes in clinical outcome measures are small and not likely to have functional implications for patients. However, because the measure may be more sensitive than patients' subjective sense of progression, it is relevant for therapy trials to detect small effects. For the MFM, a minimal clinically important difference is defined as 2.5% to 5% change.^42,43^ In our study, a change of 7.1% was seen in patients with OPMD. For the other clinical measures, no studies exist on the minimal clinically important differences. Future longitudinal studies with the focus on the minimal clinical important differences of clinical outcome measures in patients with OPMD are needed to confirm our findings.

No relationship was found between disease progression and disease duration or genotype. On the contrary, fast and slow rates of disease progression were found in both mildly and severely affected patients. There must be epigenetic or environmental factors that influence disease progression that are not known yet. Larger studies with repeated follow-up measures are needed to ascertain a pattern in progression rates.

The results of this study may be influenced by the following shortcomings. The maximum bite force was measured by a validated tool that measures the bite force with the front teeth but not with the molar teeth. Bite force may have been underestimated by this, although it does not change the value of these longitudinal data. Another limitation is that we may have left relevant measurements out of the examination, despite the carefully composed study protocol. Dynamometry measurements to assess the strength of the hamstring muscles may be relevant, but also spirometry to measure vital force capacity is suggested to be relevant in OPMD in relation to dysphagia and dysarthria.^31^ Furthermore, for 2 measures, some patients were unable to perform the task (10 patients for TOMASS chewing time and 9 patients for stair-walking test), which suggests underestimation. This could have implications for the magnitude of the differences. Furthermore, judging ophthalmoplegia on the video recordings was difficult, and progression was not possible to reliably objectify. Finally, a third time point during follow-up was not feasible within our study design but would allow more solid conclusions on the responsiveness of the outcome measures. Further research is needed to make statements on whether the clinical outcome measures can detect disease progression over a shorter or longer time frame of a therapeutic trial.

An unexpected observation was that although most patients showed disease progression in the muscle strength tests, a few patients performed better at follow-up. Some of them reported that they had developed a more active lifestyle by playing sports or having adapted a walking routine during the study period. We did not systematically ask patients about their daily activities and sports, but it would be interesting to examine whether an active lifestyle may affect muscle strength and functional capacity in patients with OPMD as it did in myotonic dystrophy type 1 and facioscapulohumeral muscular dystrophy.^44,45^

This study shows the feasibility of quantifying disease progression in OPMD within 20 months, the time frame of a drug trial. This paves the way for reliable clinical trials in humans. Equally important is that our results show that weakness of the shoulder girdle and lower limb girdle is more sensitive to changes than the oropharyngeal measurements. However, further research is needed to confirm and explain these findings in larger (international) cohorts.

## Glossary

CI: confidence interval
CRAMP: Computer Registry of All Myopathies and Polyneuropathies
INQoL: International Quality of Life
MFM: Motor Function Measure
MRC: Medical Research Council
NRRS: Normalized Residue Ratio Scale
OPMD: oculopharyngeal muscular dystrophy
SF-36: Short Form-36
TOMASS: Test of Masticating and Swallowing Solids
